# Integration of e-learning approaches in a post-pandemic learning environment – Norwegian nursing students’ recommendations from an action research study

**DOI:** 10.1016/j.heliyon.2023.e13331

**Published:** 2023-01-31

**Authors:** Karina Karlsen, Charlotte Aronsen, Therese Daleng Bjørnnes, Trine Bruun Harberg, Anita Nytræ Halland, Thomas Holand, Lena Jakobsen, Liv Kornbakk, Britt-Inger Kvalshaug, Hilde Lian, Carina Nygård, Ann Kristin Solsvik, Elisabeth Trømborg, Nina Emaus

**Affiliations:** Department of Health and Care Sciences, Faculty of Health Sciences, UiT the Arctic University of Norway, Norway

**Keywords:** Nurse students, Online learning, Digital approaches, Action research

## Abstract

The covid-19 pandemic has profoundly led to changes in use of digital platforms, online teaching, and e-learning strategies. The aim of the present study was to examine how future pedagogical approaches can promote constructive learning environments and facilitate nursing students learning processes in future post-pandemic scenarios based on an action research study, which were conducted through several steps from January 2021 through January 2022 in a Bachelor of Nursing program at UiT the Arctic University of Norway. Students from the 2020 enrollment were invited to focus group interviews in March 2021. The interviews were transcribed, and content analyzed, resulting in concrete advice, which were implemented for the next enrollment. Students from the 2021 enrollment were invited to similar focus groups, resulting in refined advice, which will be presented. The pandemic situation resulted in extensive use of digital platforms for the 2020 enrollment. Students from this cohort described shock and disappointment connected to their study start. They expected a new life, meeting new friends and to develop a student identity, but their expectations were broken. The loss of social connections led to isolation and a weak link to the program and to the nursing profession. They recommended integrated training of theoretical and practical skills in small groups combined with short, well-prepared digital lectures equivalent to “flipped classroom” approaches. Implementing their advice, the 2021 enrollment's experience became different. This group was prepared for extensive use of digital platforms and gave future advise on increased flexibility and balance between the various learning activities centered around the clinical sessions. Based on these experiences during the pandemic, we suggest that digital platforms and e-learning strategies facilitate nursing students learning in combination with active and social learning environments.

## 1 Introduction

Nursing education comprise theoretical studies and integration of theory into practical skills [1]. According to Patricia Benner it is equally important that nursing students over time are formed through personal processes during the learning activities and through exposure to professional practice [2]. During the first semester, nursing students are introduced to their future profession and most nursing studies will imply studies in anatomy, physiology and communication, and the integration into basic nursing skills. The first semester is also critical for development of learning strategies and for formation of the learning environment [3]. The covid-19 pandemic resulted in rapid and unexpected use of digital platforms and e-learning strategies in higher educational institutions worldwide, including nursing educational programs [4]. Exploring experiences of nurse educators who transitioned from traditional classroom to online learning environments, Sinacouri found that nurse educators experienced a need for learning both a new management system, technological support, as well as a need for mind shift in pedagogical approach [5]. Despite rapid changes, Wilson et al. indicate that nursing faculties have been resourceful and adaptive using both novel and existing resources and methodologies to meet their teaching objectives and engage students [4]. They also suggest that in the future, more programs and courses will continue to be managed online [4]. These changes, not previously experienced in academia, will according to Williamson et al. require an ongoing institutional commitment to meeting curricular goals and objectives while maintaining a commitment to being student focused [6]. A cross-sectional study by Vizcaya-Moreno and Perez-Canaveras identified that the most useful and preferred teaching methods during clinical nursing studies were online tutorials or videos, interactive gaming, and virtual learning environments [7]. Referring to nursing students of today as the generation Z students, they also highlighted that today's nursing students carry a distinctive combination of attitudes, beliefs, social norms, and behaviors that will modify education and the nursing profession in the future [7]. Other recent studies support the notion of preparedness and readiness among nursing students towards further development of e-learning environments [8], preferably in combination with problem-based learning strategies [9]. There are however also evidence indicating that previous experience with e-learning is an important factor for learning outcome among nursing students [10], that virtual learning during the pandemic period has caused substantial psychological stress among nursing students [11,12] and that traditional clinical practice fully replaced by screen-based simulation will result in reduced learning outcome [13]. Studies also indicate that we are in the middle of a transitional phase in nursing education that must be followed carefully [14], a situation also applied to other health professional educational programs such as medical schools [15]. With the ongoing pandemic, during 2020 and 2021, and with evolving research pointing out dilemmas and conflicting findings, we conducted an action research study among nursing students during their first semester in 2020 and in 2021. We explored their experiences during two identified phases of the pandemic, and the aim of the present study was to identify how use of digital platforms and e-learning approaches can promote constructive learning environments and facilitate nursing students learning processes in future post-pandemic scenarios.

## 2 Method

Action research is described as a useful method of studies directed towards own practices, including the field of nursing [16,17]. According to McDonnell and McNiff [18], action research is specially designed for evaluation and improvement of existing practice, including documentation of transitional phases [19]. Action research in higher education may furthermore provide a framework for development of nursing education [20]. The hallmark of action research is intervention and its democratic and participatory design [18,19]. We conducted our study through several steps over a one-year period from January 2021 through January 2022 in the bachelor nursing program at UiT the Arctic University of Norway (UiT), campus Harstad which has an annual enrollment of 75 nursing students starting in August every year. Despite a relatively low prevalence of Covid-19 cases in Norway during the first months of the pandemic from February to July 2020 [21], also Norway experienced an increase in cases as the second wave was apparent from August 2020 [22]. The Ministry of Education and Research decided that all teaching at higher institutions in Norway should be online with opportunity to collect students in small groups or cohorts, for training sessions where online sessions were no alternative. For the group of 75 enrolled nursing students at UiT campus Harstad in August 2020, the situation mainly meant digital lessons.

August 2021 came with an ongoing pandemic situation after 1.5 years, high vaccination rates in Norway, but not yet into full step four in the Government's plan for opening of Norway. UiT's recommendation included digital lectures where possible but allowed gathering of students in cohorts in classrooms where 1-m distance between participant could be obtained. Due to the conditions, some gatherings of students across disciplines were arranged. The 2021-enrollment were divided into fixed groups with approximately 10 students in each group. Theoretical lectures were mainly digital, but also “hybrid” solutions with parts of the enrollment present physically in the classroom and other parts of the group online. Clinical learning sessions were done in smaller fixed groups.

For data collection we used focus group interviews based on a predefined interview guide with open questions concerning social life, learning environment, and the study situation [[23], [24], [25]], with a maximum of 8 students in each group. Three teachers were present during the interviews, all with predefined roles: facilitator, interviewer and moderator [26]. All students from the 2020 enrollment were invited for two focus group interviews in March 2021, when they were in their second semester of the studies. Participation was open until the two preplanned groups were complete. Two interviews with altogether 15 participants were performed. We transcribed the interviews, and through a content analysis including detailed thematic coding, categorization into main topics were performed [19,27]. From these results we derived concrete advice, which was implemented for the 2021 enrollment, in an ongoing pandemic situation. Students from the 2021 enrollment were invited to similar focus groups. Due to a busy schedule one focus group interview with 6 participants was performed in September 2021, six weeks into the first semester. All participating students gave written informed consent. The Norwegian Centre for Research Data approved the project and data collection in January 2021. In the result chapter we first present the main results from the interviews with the students from the 2020-enrollment. Thereafter we describe the adjustments made for the 2021-enrollment. Finally, we outline the experiences and advise from the participating students of the 2021-enrollment. Based on our interpretation from this circular process, we provide our advice considering existing literature in the discussion chapter.

## 3 Results

### 3.1 Experiences from the 2020 enrollment

During the interviews, the students uniformly described a feeling of shock and disappointment connected to study start. They had all expected to “start a new life”, meeting new friends and had been looking forward to a new student identity, but all their expectation were broken. They deeply missed the chance to get familiar with their mates and the loss of social connections for many led to isolation and a weak link to the program and to the nursing profession. The thematic coding process resulted in three main categories: *broken expectations*, *the importance of the “human touch”* and *advice to the study program*.

### 3.2 Broken expectations

Before starting the semester, the students had been looking forward to starting a new chapter in their lives, to meet new friends and acquaintances and to start nursing studies. As one of the students said it:

“All my family and relatives had told me so much about this very special time in life and encouraged me to enjoy it and to have a lot of fun”.

Instead, the students had to stay at home and were introduced to their mates on zoom. They were divided into “various break out rooms”, which did not help them to establish close relationship with their classmates. They said:

“It was hard to get to know each other through the digital platforms”.

They felt lonely and several expressed “declining psychological health”. Those with families around collected support from their family and those living in collectives gained support from friends. Students from outside the town living alone confirmed the feeling of loneliness. They all felt very unsecure about their identity as students, and although they were used to digital media, they said:

“It was hard to become familiar with the various digital platforms, such as zoom, teams and canvas”.

### 3.3 The importance of the “human touch”

The circumstances influenced the students learning processes profoundly. Not knowing each other it was not easy to turn to each other for discussions and reflections over topics related to the study. Some expressed that they

“Just made a decision to take a strong control over their own learning process and that this made them very independent”.

Others – a majority – said they

“Struggled, lost motivation and had to battle strong urges to drop out”.

Some also expressed

“Difficulties in maintaining routines, concentration and to stay updated in the canvas room”.

Concentration was easily lost during the zoom lectures, especially during long lectures. After half a year, they feared that they had not achieved the expected professional and technical skills, and they were worried about their performance during the upcoming practice. They also expressed that they

“Missed a closer contact with the teachers”.

They strongly appreciated the involvement of the contact teacher, which helped them not to give in and encouraged all the teachers to show personal involvement also during the digital sessions.

### 3.4 Advice to the study program

In unison, the students advised us to divide the next batch of students into fixed groups from the first day and with teachers available to help the groups to become well established. Courses on digital platform use should be available for the students. If the pandemic should continue, they recommended

“As much physical attendance as possible and priority to social activities and gatherings”

They all agreed that physical clinical learning sessions preparing them to the nursing profession should be given the highest priority. The students strongly recommended

“Structured reading plans within each theoretical subject”

If physical lectures still should be impossible, short digital lectures to be followed up by physical seminars, preferably in the fixed groups were recommended. Furthermore, they pointed out that

“*Numbers of teachers involved should be reduced to a minimum, because it is not easy for the students to sort the teachers from each other, and to become familiar with the responsible teachers that they can turn to in case of questions”*

The teachers should, both during lectures and seminars work hard to involve the students, and use various means, like polls etc. to engage them and keep them active – because it easy to “hide oneself” and keep a passive profile during digital sessions. One-way communication should be reduced to a minimum and lectures including two teachers promote activity. On questions about use of camera, the students encouraged teachers to recommend camera use, not to request it. They also recommended teachers

“To learn from each other and to share ideas and good online lecturing practice”

Students should be asked to follow the chat-function – and teachers should stick strictly to the schedule. Furthermore, both groups highlighted that there are many advantages with digital sessions:

*“It gives us the opportunity to participate from anywhere, and we can save valuable traveling time. So therefore, used with caution, we really recommend it”*.

### 3.5 Experiences from the 2021 enrollment

Planning the timetable for the 2021-enrollment, we tried as much as possible to follow up on all the points provided. The overall impression from the group interview with the students from the 2021 enrollment is a totally different atmosphere. They all express that

“The start of the semester has been so much better than expected”.

Generally, they have enjoyed the campus gatherings, meetings with their classmates and they all express a feeling of joy and excitement over being a student and in particular a nursing student. The identified categories from the interview were that *social life promotes learning but requires energy* and *balancing learning activities*.

### 3.6 Social life promotes learning but requires energy

Starting the semester, the students were eager to meet other people and they expressed

“A huge desire for social life and for someone to discuss study topics with”.

They also expressed a strange feeling of knowing one's own group well and less the rest of the class. Therefore, the campus gatherings and the few gatherings including the whole enrollment were very much appreciated and made them “feel like real students”. Some also express a feeling of nervousness:

“I enjoyed being together with my peers very much, but I was afraid that I would bring home this disease”.

For those with family close, this meant that they chose not to participate in the campus gatherings, something which made especially those feel *“a bit outside the group”* and unsecure in the student role in the beginning weeks. Uniformly they all expressed that

“The getting to know each other better activities made me feel more secure, it made it much easier to participate in discussions in the allocated group and to take a more active role during the digital sessions”.

Interestingly, the students clearly expressed that

“Socializing made me so tired, in a way that I had not experienced before”

This made them all more conscious about the balance between being social and *“my personal need for being alone and on my own”*. This made them also more aware about their own need for sleep, especially after demanding study weeks.

### 3.7 Balancing learning activities

The impression from the interview is that the group of students were generally satisfied with the structure of the study start and their reflection is very much connected to how the study best should be structured in a future post-pandemic reality. They clearly expressed an advice of continuous pedagogic development based on lessons learnt. Their primary focus was on “*balance and flexibility”* between a) hours spent at school and hours during the day for their own disposal c) digital, hybrid and physical lectures d) fixed and randomly designed groups. The clinical sessions constituted the central part of their study, and they acknowledged that these sessions must be organized through fixed groups. Furthermore, they recommended a much closer link between the clinical sessions and anatomy and communication section:

“If we practice proper bed rest at the same week as we study the heart and lung if will help us to understand the circulatory system and how to communicate with a seriously seek person”.

They also recommended physical theoretical lectures:

“Generally, the teachers are much more inspiring during the physical lectures, and it is much easier to be engaged and to ask questions”.

But they all agreed that digital participation must remain an option also in the future:

“*Although I was in quarantine, I could still keep up my studies and without digital participation I would for sure have dropped out”.*

They also based this advice on the need for *“balance between social life and time on my own”*. As one of the students expressed it:

“Last week I participated in the morning lectures from home and went down to the campus for the group sessions in the afternoon, this flexibility makes studying much easier for me”.

They also expressed a clear expectation of future teachers to be familiar with the use of various options of the digital platforms that promote learning goal achievements such as using polls, break out-rooms, videos etc.

## 4 Discussion

This action research study with students enrolled in a Bachelor of Nursing program during the Covid-19 pandemic illustrates the sudden shift in teaching methods that neither students nor teachers were prepared for [4,14,15]. Our findings indicate that the nursing students in the 2020-enrollment – half a year into the pandemic – experienced the toughest changes and that the circumstances seriously affected their well-being and motivation for studying, as indicated by others [11,12,28]. The 2020 restrictions connected to social gatherings also had an impact on their professional identity development – as students and especially on their identity as nursing students [[29], [30], [31]]. The restrictions influenced the formational process described by Benner [2] leaving them in doubt about their professional choice. Despite an ongoing pandemic situation, students in the 2021-enrollment were offered physical gatherings on campus with their classmates with distance keeping as one important restriction. Having lived one and a half year into the pandemic, it is our interpretation that they were much better prepared for extensive use of digital platform, resulting in less frustrations, which recent research also have indicated [10].

In an interesting paper from 2020 Vizcaya-Moreno and Perez-Canaveras characterize today's nursing students as the generation Z nursing students who will, with their expectations, attitudes and beliefs modify nursing education and the nursing profession in the future [7]. In their study the identified most useful and preferred teaching methods during clinical nursing studies were online tutorials or videos, interactive gaming, and virtual learning environments [7]. These findings corresponded Hampton et al. [32] who pointed out that assigned reading was the least preferred method of learning for the generation Z nursing students, rating traditional classrooms teaching with the use of various methods for learning stimulation and student engagement very high. In addition, based on our findings, we should expect that future nursing students will register with an expectation that learning activities actively will include the use of digital platforms. This was pointed out by Williamson and Muckle already in 2018 [33], arguing that since technology has become such an integral part of a nurse's practice, use of technology should be integrated into the nursing curriculum and teaching environment [33]. In a paper from 2021 Williamson et al. [6] claimed that the global Covid-19 pandemic has triggered changes in nursing education at a pace not previously experienced. In line with our results, they also highlighted the challenges of balancing digital as well as face-to-face classrooms, laboratory and clinical learning, at the same time promoting learning outcomes and demanded professional competencies [6]. They suggested that use of transformational change theory may facilitate and support ongoing institutional commitment worldwide to creating student focused learning environments within this new area [6], a notion we strongly support.

According to Benner [2], nursing students are formed through personal changes during the learning processes, activities and through exposure to professional practice. Benner recommends engaged situated reasoning and the use of practice situations as basis for learning to become a skilled and professional nurse [1]. The recommendations given by the students from the 2021-enrollment are in line with the thinking offered by Benner. For the students, the central learning arena identified was the clinical sessions, which offered them a strong sense of studying bed-side nursing (enhanced formation), and they recommended that the learning activities should revolve around the clinical sessions with extensive use of e-learning platforms in all other learning activities.

The strength of the present action research study was the opportunity to interview students in midst of a pandemic situation with ongoing introduction to the use of digital platforms. We focused on the first critical semester of a Bachelor of nursing program, and we used students' advice to change our pedagogic approach. All changes were implemented in an ongoing pandemic situation. Being in what is hopefully the end, we think that our findings and experiences are relevant for a post-pandemic situation which will involve a successful integration of e-learning strategies, as supported by many researchers today [6]. There are limitations of the study. We interviewed students from the 2020 enrollment in March 2021, three months after finishing their first semester. The interview data was therefore based on their memory and not on their direct experiences. However, they all seemed to remember this critical period of their life very well. We only managed to conduct one focus group interview with students from the 2021 enrollment. This was however a very rich interview where the students spoke very openly about their experiences. We therefore feel that contrasting the 2020- and the 2021-students experience is valid. We are aware that we have explored students’ experiences and not their learning outcomes. However, present evidence indicates that learning environment profoundly influences motivation for learning and learning goal achievement [34,35]. Halverson [36] uses the picture of a storm on sailing and the need to navigate wisely within the realm of uncertainty, and our study support the notion of being at a time to move forward with new ideas [37]. There is a need – and an opportunity - to implement changes that will enhance students' learning and support their mental health in a post pandemic nursing education at the same time as we facilitate their identity as nurses, scholars, and leaders who can deal effectively with change and uncertainty [37].

## 5 Conclusion

Based on the experiences and lessons learnt during the pandemic, our study indicate that future nursing students will expect integration of digital platforms and e-learning strategies in combination with active and social learning environments centered around clinical sessions as pedagogic strategies. Today's nursing students are well prepared for such strategies already from the first semester.

## Declarations

### Author contribution statement

Karina Karlsen; Charlotte Aronsen; Liv Kornbakk: Conceived and designed the experiments; Performed the experiments; Wrote the paper.

Therese Dalen Bjørnnes; Trine Bruun Harberg; Anita Nytræ Halland; Thomas Holand; Lena Jakobsen; Britt-Inger Kvalshaug; Hilde Lian; Carina Nygård; Ann Kristin Solsvik; Elisabeth Trømborg; Nina Emaus: Conceived and designed the experiments; Wrote the paper.

### Funding statement

The study was supported by the Department of Health and Care Sciences at UiT the Arctic University of Norway.

### Data availability statement

Anonymous transcribed files are available in Norwegian upon request.

### Declaration of interest’s statement

The authors declare no competing interests.

## References

[1] Benner P. (2012). Educating nurses: a call for radical transformation-how far have we come?. J. Nurs. Educ..

[2] Benner P. (2011). Formation in professional education: an examination of the relationship between theories of meaning and theories of the self. J. Med. Philos..

[3] Flott E.A., Linden L. (2016). The clinical learning environment in nursing education: a concept analysis. J. Adv. Nurs..

[4] Wilson J.L. (2021).

[5] Sinacori B.C. (2020). How nurse educators perceive the transition from the traditional classroom to the online environment: a qualitative inquiry. Nurs. Educ. Perspect..

[6] Williamson K.M. (2021). Opportunities in chaos: leveraging innovation to create a new reality in nursing education. Nurs. Adm. Q..

[7] Vizcaya-Moreno M.F., Perez-Canaveras R.M. (2020). Social media used and teaching methods preferred by generation Z students in the nursing clinical learning environment: a cross-sectional research study. Int. J. Environ. Res. Publ. Health.

[8] Alhassan R.K. (2020). Assessing the preparedness and feasibility of an e-learning pilot project for university level health trainees in Ghana: a cross-sectional descriptive survey. BMC Med. Educ..

[9] Ding Y., Zhang P. (2018). Practice and effectiveness of web-based problem-based learning approach in a large class-size system: a comparative study. Nurse Educ. Pract..

[10] Alqahtani N., Innab A., Bahari G. (2021). Virtual education during COVID-19: exploring factors associated with E-learning satisfaction among Saudi nursing students. Nurse Educat..

[11] Amerson R. (2021). Nursing education amid a pandemic: mental health in a time of virtual learning. Nurse Educat..

[12] Nodine P.M. (2021). Graduate nursing student stressors during the COVID-19 pandemic. J. Prof. Nurs..

[13] Leighton K. (2021).

[14] Jallad S.T., Isik B. (2021). Transitioning nursing students' education from traditional classroom to online education during the COVID-19 pandemic: a case study applied to the meleis trial. Flor. Nightingale J. Nurs..

[15] Dost S. (2020). Perceptions of medical students towards online teaching during the COVID-19 pandemic: a national cross-sectional survey of 2721 UK medical students. BMJ Open.

[16] Sullivan E., Hegney D.G., Francis K. (2013). An action research approach to practice, service and legislative change. Nurse Res..

[17] Greenwood J. (1994). Action research and action researchers: some introductory considerations. Contemp. Nurse.

[18] (2017). Action research for nurses McDonnell peter and McNiff jean action research for nurses 192pp pound24.99 sage 9781473919402 1473919401 [formula: see text]. Nurse Res..

[19] Meyer J. (2000). Qualitative research in health care. Using qualitative methods in health related action research. BMJ.

[20] Moch S.D. (2016).

[21] Yarmol-Matusiak E.A., Cipriano L.E., Stranges S. (2021). A comparison of COVID-19 epidemiological indicators in Sweden, Norway, Denmark, and Finland. Scand. J. Publ. Health.

[22] James N., Menzies M., Radchenko P. (2021). COVID-19 second wave mortality in Europe and the United States. Chaos.

[23] Nyamathi A., Shuler P. (1990). Focus group interview: a research technique for informed nursing practice. J. Adv. Nurs..

[24] Rabiee F. (2004). Focus-group interview and data analysis. Proc. Nutr. Soc..

[25] Basch C.E. (1987). Focus group interview: an underutilized research technique for improving theory and practice in health education. Health Educ. Q..

[26] McLafferty I. (2004). Focus group interviews as a data collecting strategy. J. Adv. Nurs..

[27] Krueger R.A. (2006). Analyzing focus group interviews. J. Wound, Ostomy Cont. Nurs..

[28] Mulyadi M. (2021). Prevalence of mental health problems and sleep disturbances in nursing students during the COVID-19 pandemic: a systematic review and meta-analysis. Nurse Educ. Pract..

[29] Cook T.H., Gilmer M.J., Bess C.J. (2003). Beginning students' definitions of nursing: an inductive framework of professional identity. J. Nurs. Educ..

[30] Williams M.G., Burke L.L. (2015). Doing learning knowing speaking: how beginning nursing students develop their identity as nurses. Nurs. Educ. Perspect..

[31] Goodolf D.M. (2018). Growing a professional identity: a grounded theory of baccalaureate nursing students. J. Nurs. Educ..

[32] Hampton D., Welsh D., Wiggins A.T. (2020). Learning preferences and engagement level of generation Z nursing students. Nurse Educat..

[33] Williamson K.M., Muckle J. (2018). Students' perception of technology use in nursing education. Comput. Inform. Nurs..

[34] Rojas Reyes J., Rivera Alvarez L.N., Morera Pomarede M.J. (2018). Pedagogic aspects in nursing education: integrative review. Invest. Educ. Enfermería.

[35] Gronlien H.K. (2021). A blended learning teaching strategy strengthens the nursing students' performance and self-reported learning outcome achievement in an anatomy, physiology and biochemistry course - a quasi-experimental study. Nurse Educ. Pract..

[36] Halverson K.L. (2021). Sailing in the winds of change: navigating the future of nursing education. J. Nurs. Educ..

[37] Valiga T.M. (2021). Postpandemic nursing education: moving forward with new ideas. J. Nurs. Educ..

